# Patterns of GPS measured time outdoors after school and objective physical activity in English children: the PEACH project

**DOI:** 10.1186/1479-5868-7-31

**Published:** 2010-04-22

**Authors:** Ashley R Cooper, Angie S Page, Benedict W Wheeler, Melvyn Hillsdon, Pippa Griew, Russell Jago

**Affiliations:** 1 Department of Exercise, Nutrition and Health Sciences, University of Bristol, Tyndall Avenue, Bristol BS8 1TP, UK; 2 Peninsula College of Medicine & Dentistry, Knowledge Spa, Royal Cornwall Hospital, Treliske, Truro, Cornwall, TR1 3HD, UK; 3 School of Sport and Health Sciences, University of Exeter, St Luke's Campus, Heavitree Road, Exeter EX1 2LU, UK

## Abstract

**Background:**

Observational studies have shown a positive association between time outdoors and physical activity in children. Time outdoors may be a feasible intervention target to increase the physical activity of youth, but methods are required to accurately measure time spent outdoors in a range of locations and over a sustained period. The Global Positioning System (GPS) provides precise location data and can be used to identify when an individual is outdoors. The aim of this study was to investigate whether GPS data recorded outdoors were associated with objectively measured physical activity.

**Methods:**

Participants were 1010 children (11.0 ± 0.4 years) recruited from 23 urban primary schools in South West England, measured between September 2006 and July 2008. Physical activity was measured by accelerometry (Actigraph GT1M) and children wore a GPS receiver (Garmin Foretrex 201) after school on four weekdays to record time outdoors. Accelerometer and GPS data were recorded at 10 second epochs and were combined to describe patterns of physical activity when both a GPS and accelerometer record were present (outdoors) and when there was accelerometer data only (indoors). ANOVA was used to investigate gender and seasonal differences in the patterns of outdoor and indoor physical activity, and linear regression was used to examine the cross-sectional associations between GPS-measured time outdoors and physical activity.

**Results:**

GPS-measured time outdoors was a significant independent predictor of children's physical activity after adjustment for potential confounding factors. Physical activity was more than 2.5 fold higher outdoors than indoors (1345.8 ± 907.3 vs 508.9 ± 282.9 counts per minute; F = 783.2, p < .001). Overall, children recorded 41.7 ± 46.1 minutes outdoors between 3.30 pm and 8.30 pm, with more time spent outdoors in the summer months (p < .001). There was no gender difference in time spent outdoors. Physical activity outdoors was higher in the summer than the winter (p < .001), whilst there was no seasonal variation in physical activity indoors.

**Conclusions:**

Duration of GPS recording is positively associated with objectively measured physical activity and is sensitive to seasonal differences. Minute by minute patterning of GPS and physical activity data is feasible and may be a useful tool to investigate environmental influences on children's physical activity and to identify opportunities for intervention.

## Background

Children who are physically active are less likely to be overweight [1] and have better cardiovascular risk factor profiles [2] and other indicators of health [3] than less active children. There is widespread concern that children's physical activity has been declining over recent decades, and that this may underpin population level increases in childhood overweight and obesity as well as creating a potential burden of future ill health. These concerns have led to policies to increase children's physical activity in many countries, and increased interest in studying the determinants of children's physical activity in order to develop effective interventions. Of particular interest is the role that physical environmental factors may play in influencing young people's physical activity. At present, relatively little is known about which aspects of the environment may influence young people's physical activity and research in this area is limited by predominantly cross-sectional study designs and limited use of objective methodologies [4].

One potential environmental influence on children's physical activity is the amount of time spent outdoors. Time outdoors has been found to be a correlate of children's physical activity in most studies [5,6] and has been associated with higher objectively measured physical activity [7,8], with lower prevalence of overweight [8] and with higher independent mobility [9]. It has been suggested to be a mediator between neighbourhood greenness and BMI [10] and as a proxy measure for children's physical activity [11]. Encouraging children to spend more time outdoors may thus be an effective strategy for increasing physical activity and preventing overweight and obesity [8]. In order to evaluate such an intervention, a methodology is required to accurately measure time spent outdoors in a range of locations and over a sustained period of time. In previous studies, time outdoors has been estimated indirectly by parental proxy [8,12] or participant self-report/diary [9], methods which are subject to a lack of precision and potential reporting bias, or by direct observation which provides an accurate estimate but is labour intensive and limited to relatively small samples observed in defined locations [7].

The Global Positioning System (GPS) is now being used to describe how individuals interact with the physical environment. The development of lightweight personal GPS receivers allows the outdoor location of an individual to be recorded with high frequency and accuracy, and combining GPS data with objectively measured physical activity has the potential to enable both the location and use of physical activity promoting or inhibiting environments to be described. At present very few studies have used this technology in free living children. Personal GPS receivers have been used to map travel routes to school [13,14] and combined accelerometer and GPS data have been used to describe associations between children's physical activity and independent movement [15], travel to school [16] and the location of bouts of moderate intensity physical activity [17]. GPS tracking of individuals produces a data set that is highly detailed but which is also highly complex to interpret, and most studies have been limited to a small number of people or to specific behaviours (e.g. the journey to school), occurring within a prescribed period of time.

GPS receivers record data predominantly when outdoors, and a potential use of GPS that does not require mapping of the location of participants may be to provide a measure of time spent outdoors. The aim of this study was to investigate the association between GPS measured time outdoors and objectively measured physical activity among a large sample of free-living primary school-age children.

## Methods

### Participants

This paper reports cross-sectional data from the baseline sample of 1307 children recruited for the PEACH (Personal and Environmental Associations with Children's Health) project, a UK longitudinal study investigating the environmental and personal determinants of physical activity in children across the transition from primary to secondary school. Children in their last year of primary school (Year 6) were recruited from 23 state schools within a UK city. The primary schools were selected as those with transition rates >40% to one of eight urban state funded secondary schools which were chosen to be representative of the city on the basis of geographic location and the Index of Multiple Deprivation (IMD) [18,19]. Only one primary school of those approached declined to take part in the study. Baseline data were collected between September 2006 and July 2008. A University Ethics Committee approved the study and written informed consent was obtained from a parent/guardian of all participating children.

### Measures

Physical activity was measured every 10 seconds using an accelerometer (GT1M; ActiGraph LLC, FL, USA) worn on a belt around the waist during waking hours. Positional data were recorded at 10 second intervals using a GPS receiver (GPS; Garmin Foretrex 201) [20]. Children were trained to switch the GPS on and off to conserve battery life and were asked to wear it on either wrist after school on four consecutive school days. "Month" of data collection was defined by the first day of data collection for each participant. Similarly the first day of data collection was used as the reference point for daylight hours and the mean minutes of daylight from 3 pm until sunset on that day were obtained standard tables. "Season" (summer/winter) was defined by British Summer Time (BST), when clocks go forward (late March) or back (late October) by 1 hour respectively. Height (m) was measured with a stadiometer and weight (kg) was measured using digital scales (SECA), with children wearing indoor clothing, and shoes removed. Body Mass Index (BMI = kg/m^2^) was calculated and UK age and gender specific BMI Standard Deviation Scores (SDS) were derived from standard tables [21]. Pubertal status was measured using the Petersen scale [22] and five derived stages (equivalent to Tanner stages) were used in analyses.

### Data processing & statistical analysis

Raw accelerometer files were downloaded using ActiLife v1.0.52 (ActiGraph LLC) and imported into Stata/IC v10 (Stata Corp. College Station, TX, 2007). Periods of ≥60 minutes of zero values were defined as accelerometer "non-wear" time and discarded. GPS data were downloaded to GPS Utility v4.9.2 (GPS Utility Ltd, Southampton, 2007), and date and time matched to the accelerometer data using script written within Stata. To allow time matching, the start time of each GPS epoch was rounded down to match the start time of each accelerometer epoch (e.g. a GPS measure at 08:43:47 would be matched to the accelerometer epoch starting at 08:43:40). Data recorded when the participant was travelling at ≥15 kph were discarded as being in motorised transport (10% of data). In this paper we have defined accelerometer data matched to a GPS record as "physical activity outdoors" and unmatched accelerometer data (no GPS record) as "physical activity indoors" since the GPS receiver used in this study does not record positional data when inside a building.

Data were initially recorded at 10s epochs and were collapsed to provide minute by minute data. Accelerometer data were summed to provide accelerometer counts per minute (cpm) and each minute was coded as "outside" (matched accelerometer and GPS data) or "inside" (accelerometer with no matching GPS record). Only data recorded between 3.30 pm and 8.30 pm (defined as "after school") were used in this study since very little GPS data were recorded outside of these hours. For inclusion in the analyses children were required to have recorded at least 3 hours of accelerometer data in this period on at least one of the four measurement days.

#### Analysis

The intra-class correlation across measurement days was used to examine the reliability of total measured GPS time outdoors over 4 days. Values were strongly correlated (ICC = 0.74, p < .01) across the four days of measurement and associations with physical activity were consistent (data not shown) and thus data from all days were combined. GPS time outdoors data were log_10 _transformed to achieve a normal distribution. However, since there were no differences in analyses using transformed or untransformed data, untransformed values are shown in the text for clarity. Analysis of variance tests were used to investigate differences in mean physical activity indoors/outdoors and in physical activity and time outdoors between gender, season, and month. Linear regression models were used to examine the associations between physical activity and time outdoors, with models first run unadjusted and then adjusted for potential confounders (gender, age, BMI SDS, IMD, daylight, pubertal status). As the children were recruited from schools the model was also adjusted for the clustering of participants within schools, and robust standard errors were used. All data were analysed using Stata/IC v10 in 2009/10. Significance was set at p < 0.05.

## Results

### Participants

Of 1899 children invited to take part, 1340 provided parental consent (70.5%) and 1307 were present in school on measurement days. Thirty-three accelerometers were not returned or were broken, 8 files could not be read, 12 contained no data and 25 had a timing fault and could not be matched with the GPS. Of the remaining 1229 children, 151 did not provide any GPS data and a further 68 did not provide at least 3 hours of data between 3.30 pm and 8.30 pm on 1 or more days. The final sample was 1010 children (11.0 ± 0.4 years, 46.7% male) who provided matched GPS and ActiGraph data on at least one measurement day. Demographics (age, height, weight, BMI and physical activity) for the final sample did not significantly differ from excluded participants.

### After school-pattern of time outdoors

**Figure 1:**
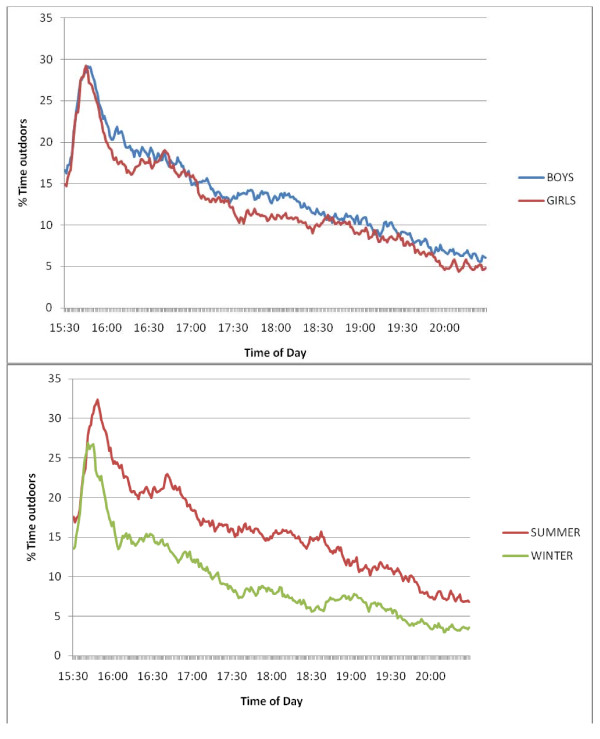
**Mean percentage of time spent outdoors between 3.30 pm and 8.30 pm by gender (upper panel) and season (lower panel)**.

The minute-by-minute pattern of time outdoors after school is shown in figure 1. School ended at approximately 3.30 pm, and the level of recording increases as the GPS receiver connects with the satellite network when children leave the school buildings. Time outdoors peaks shortly after the children leave school and is at its highest between 3.30 and 4 pm when the children were travelling home. Time outdoors then declined throughout the evening. Patterns for boys and girls were highly similar with girls recording slightly less time outdoors than boys. In the winter months when there are fewer hours of daylight and poorer weather, children are likely to spend less time outdoors. We investigated this by comparing data collected during British summer time (BST) with data collected the rest of the year (Figure 1 lower panel). Data collected in both seasons followed the same pattern, but as predicted the proportion of time spent outdoors was substantially lower in winter.

Over 90% of children recorded accelerometer data throughout the evening indicating compliance with the study protocol, whilst the number providing data outdoors declined as the evening progressed (Table 1). Overall children spent just over 40 minutes outside after school each day (41.7 ± 46.1 minutes) with the mean time outdoors highest in the first hour after school (14.1 ± 11.4 minutes) and declining through the evening (Table 1). Over half of the children (51.4%) recorded less than 30 minutes per day outdoors with 25.4% recording 30-60 minutes, 13.7% recording 60-90 minutes and only 9.6% recording over 90 minutes. Boys were outdoors slightly more than girls (43.3 ± 48.1 vs 40.2 ± 44.3 minutes) but this difference was not statistically significant. In the summer children spent more time outdoors than in the winter months (49.7 ± 50.8 vs 32.1 ± 37.8 minutes; F = 91.25, p < .001). Approximately one-third of children recorded less than 30 minutes per day outdoors in the summer compared with two-thirds in the winter (39.9% vs 64.2%) with 14.3% spending over 90 minutes outdoors in the summer (vs 4.4% in the winter).

**Table 1:** Time outdoors and physical activity levels indoors and outdoors after school in primary-school children

	3.30 pm-4.30 pm	4.30 pm-5.30 pm	5.30 pm-6.30 pm	6.30 pm-7.30 pm	7.30 pm-8.30 pm	3.30 pm-8.30 pm
n wearing accelerometer (% of total)	1002 (99.2)	991 (98.1)	995 (98.5)	970 (96.1)	923 (91.4)	1010 (100)
n providing outdoor data (% of total)	916 (90.7)	734 (72.7)	627 (62.1)	511 (50.6)	362 (35.8)	1010 (100)
Minutes outdoors*	14.1 (11.4)	10.6 (12.6)	8.12 (11.2)	6.4 (10.4)	4.4 (9.2)	41.7 (46.1)
Physical activity outdoors (cpm)	**1431.1 (946.7)**	**1401.2 (1505.7)**	**1324.5 (1660.3)**	**1204.0 (1206.7)**	**965.9 (1042.8)**	**1345.8 (907.3)**
Physical activity indoors (cpm)	**608.7 (431.7)**	**541.0 (528.9)**	**499.8 (480.5)**	**494.7 (447.2)**	**387.5 (384.4)**	**508.9 (282.9)**

Bold text = significant difference in accelerometer cpm outdoors vs indoors (p < .001). All values are (mean (sd)) unless otherwise stated.

*mean value for total n wearing the accelerometer

### GPS measured time outdoors and physical activity

**Figure 2:**
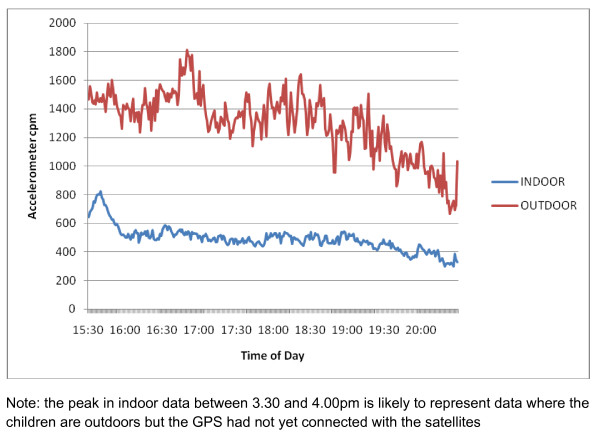
**Minute-by-minute physical activity levels indoors and outdoors between 3.30 pm and 8.30 pm**.

**Figure 3:**
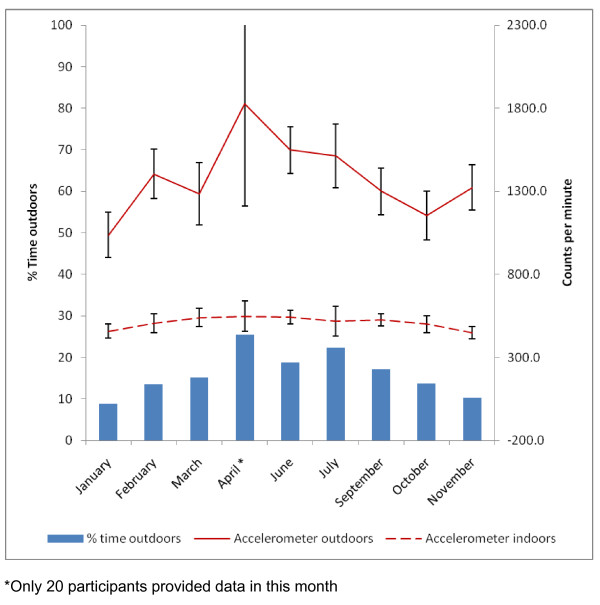
**Mean percentage of time outdoors and accelerometer counts per minute (± 95% confidence interval) outdoors and indoors between 3.30 pm and 8.30 pm by month of data collection**.

Previous studies investigating the association between time outdoors and physical activity have used overall measures of both physical activity and time outdoors. However the combination of highly time resolved GPS and accelerometer data allows both the volume and pattern of physical activity indoors and outdoors to be investigated. Figure 2 shows that minute-by-minute physical activity levels both indoors and outdoors declined throughout the evening, but that physical activity levels outdoors were consistently higher than indoors. Overall, counts per minute recorded outdoors were nearly three times as high as indoors (1345.8 ± 907.3 vs 508.9 ± 282.9 cpm; F = 783.2, p < .001) (Table 1) and this difference was consistent in each hour of measurement. Similar to the amount of time spent outdoors, overall physical activity was also higher in the summer months (665.6 ± 317.7 vs 558.2 ± 315.4 cpm; F = 28.99, p < .001). To explore this association further, accelerometer data were partitioned to be either indoors or outdoors, and were plotted against season of data collection (Figure 3). The proportion of time outdoors after school showed seasonal variation, being higher in the summer months than in the winter. Physical activity outdoors was substantially higher each month (p < .001) than that recorded indoors, and showed a marked seasonal variation. In contrast, physical activity indoors remained at a consistent level throughout the year.

In linear regression analyses, volume of time outdoors after school was a significant predictor of physical activity both in unadjusted models and after adjustment for potential confounders (gender, age, BMI SDS, IMD, daylight, pubertal status) (Table 2). Models were run for each hour to investigate whether the association varied by time of the evening and showed a consistent relationship throughout. Time outdoors was a strong predictor of physical activity when models were also run separately by gender and season (p < .001 in all models; data not shown).

**Table 2:** Linear regression of time outdoors after school and physical activity (counts per hour).

	Beta (95% CI)	t	*p*	R2
**Model 1**				
3.30 pm-4.30 pm	900.1 (703.1,1097.2)	9.48	<.001	0.144
4.30 pm-5.30 pm	1117.1 (894.3,1339.9)	10.4	<.001	0.141
5.30 pm-6.30 pm	993.4 (789.8,1197.0)	10.12	<.001	0.110
6.30 pm-7.30 pm	915.3 (685.5,1145.2)	8.26	<.001	0.108
7.30 pm-8.30 pm	715.3 (482.1,948.6)	6.36	<.001	0.064
After school (3.30 pm-8.30 pm)	1018.1 (875.4,1160.7)	14.8	<.001	0.137
				
**Model 2**				
3.30 pm-4.30 pm	871.9 (677.3,1066.5)	9.29	<.001	0.163
4.30 pm-5.30 pm	1115.3 (868.1,1362.5)	9.36	<.001	0.147
5.30 pm-6.30 pm	942.2 (714.4,1170.1)	8.58	<.001	0.125
6.30 pm-7.30 pm	901.5 (670.5,1132.5)	8.09	<.001	0.114
7.30 pm-8.30 pm	678.8 (451.8,905.9)	6.20	<.001	0.077
After school (3.30 pm-8.30 pm)	1000.6 (850.0,1151.2)	13.78	<.001	0.144

Model 1: Unadjusted

Model 2: Adjusted for gender, age, BMI SDS, IMD, daylight, pubertal status.

Beta = mean increase in counts per hour associated with a 1 minute increase in time outdoors

CI = confidence interval

## Discussion

This study investigated the volume and patterns of GPS data recorded after school, defined as time outdoors, and the association with objectively measured physical activity in primary school-aged children. Minute-by-minute plots of time outdoors showed little difference between the genders, but marked differences between the summer and winter seasons. Matching accelerometer data with GPS data allowed activity to be segmented into "indoors" and "outdoors" with high resolution. These data showed that physical activity was 2-3 fold higher outdoors than when indoors and that the level of physical activity indoors was consistent through the year whilst physical activity outdoors was seasonally patterned. Linear regression showed that GPS-measured time outdoors after school was a significant predictor of physical activity in this period. These data are consistent with previous research which has shown that time outdoors measured by self/proxy report or by direct observation [7,8] is a significant predictor of children's physical activity, and suggest that GPS recording may provide a novel method to measure time outdoors in free-living young people.

The associations described in the present study are similar to previous reports. The time spent outdoors in the spring/summer by children in this UK sample is similar to reported values for children measured during springtime in Sydney, Australia, with 40% of UK children recording <30 minutes outdoors each day compared with 37% of similarly aged Australian children [9]. In contrast, though the figures are not directly comparable, children in the present study appear to spend approximately half the time outdoors in the summer as children from Melbourne with similar though lower times outdoors in the winter [8]. The reasons for the discrepancies are unclear, but it is possible that time outdoors was differently reported in the self report/parental proxy measures used in Australia, and also possible that time outdoors is under estimated by the GPS due to the methodological issues discussed below. Further validation studies are required to resolve these discrepancies. In agreement with other studies, boys spent more time outdoors than girls, though differences were very small [7,8], and the duration of time outdoors was significantly lower in the winter months than in the summer. Combining GPS and accelerometer data enabled us to show that whilst physical activity outdoors was lower in the winter than the summer, physical activity indoors remained at a constant (lower) level throughout the year.

The period after school is an important time for children to be physically active [23,24] and the hours between 3:30 pm and 6 pm on weekdays have been described as the "critical window" for children's physical activity [8]. Accelerometry has been used to demonstrate differences in the patterns of objectively measured physical activity in this period between groups of children based on travel behaviour [25] or weight status [26], but potential environmental determinants of these differences have yet to be determined. Interpreting data from this period is complex since children leave school at variable times depending on participation in after school activities and have a wide range of possible activity patterns which may be influenced by a range of physical and social factors. The consistent association between time spent outdoors and physical activity in previous studies of primary school-aged children, and the association between proxy-reported time outdoors and weight status, indicate the importance of this measure for understanding children's physical activity. This paper supports the concept that time outside is associated with increased physical activity and that a GPS monitor potentially provides an objective method of measuring time outside. Combining accelerometer and GPS data has potential to describe associations of time spent outdoors with physical activity in different populations and locations, and also to enable the measurement of the effect of interventions aimed at increasing time outdoors. GPS technology is rapidly evolving with the development of smaller receivers built as part of other consumer products such a mobile phones, and it is likely that GPS receivers will soon be integrated with accelerometers to provide a small, single instrument for recording both activity and location. The development of standard methodologies for the combination and interpretation of these data, particularly given the episodic nature of GPS data, will greatly aid such studies.

### Strengths and limitations

This study had a number of strengths. The large sample of children was recruited to be representative of the city, encompassing a range of physical and social environments. In addition there was good compliance with wearing GPS receiver, with 80.7% of children who provided accelerometer data also providing GPS data on at least 1 day and 65% of children providing data on more than one day. However there are also a number of limitations. We defined any epoch with a GPS record as being outdoors since the GPS receiver used in this work is unable to receive a signal from the satellite network within a building unless in close proximity to a window. The signal is rapidly lost when moving into the building and whilst a large majority of GPS data are likely to be recorded when the participant is outdoors, we cannot rule out that some data may be recorded indoors. In addition, GPS data are only recorded when the instrument is switched on and connected to the satellite network. Children were trained to switch the instrument on and off, and were asked to turn it off at the end of the day to preserve battery life, turning it on again when leaving school. It is possible that some children may have failed to switch the instrument on when leaving school and that time outdoors may therefore be underestimated. Figure 2 shows a small peak of higher physical activity after school in data identified as "indoors" which is likely to be data recorded outdoors but where the GPS had not connected to the satellites or was not on. The extent to which this has happened is unknown and may result in accelerometer data defined as "indoors" actually being recorded outdoors. However, since the impact of this would be to attenuate the difference between physical activity indoors and outdoors, this would only serve to strengthen the associations reported here.

## Conclusions

Duration of GPS recording, interpreted here as a measure of time outdoors, is positively associated with objectively measured physical activity and is sensitive to seasonal differences. Public health interventions to increase the physical activity of young people may be directed towards enabling more time spent outdoors, and GPS-measured time outdoors may be a useful tool to provide a better understanding of environmental influences on children's physical activity. Further studies are required to validate GPS against other methods of assessing time outdoors.

## Competing interests

The authors declare that they have no competing interests.

## Authors' contributions

AC and AP conceived and led the study. PG co-ordinated data collection. BW matched the accelerometer and GPS data. AC conducted data analyses and wrote the initial manuscript. All authors contributed to the interpretation of data and helped in writing the manuscript. All authors read and approved the final manuscript.
